# Supplementary figures and images for: Pervasive translation in Mycobacterium tuberculosis
Source: eLife. 2022 Mar 28;11:e73980. doi: 10.7554/eLife.73980 (PMC9094748; doi:10.7554/eLife.73980)

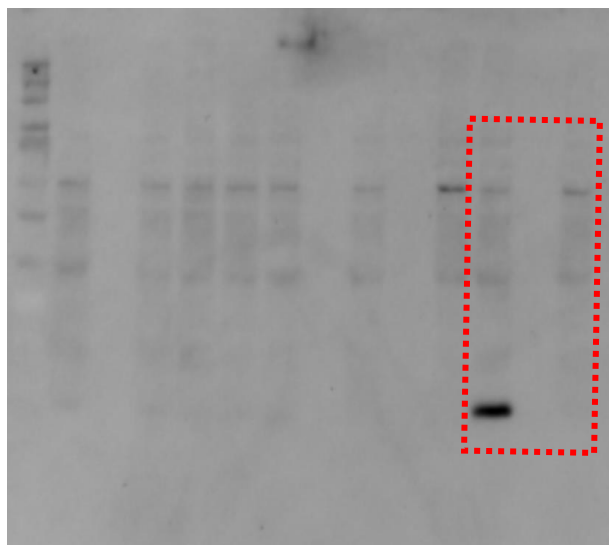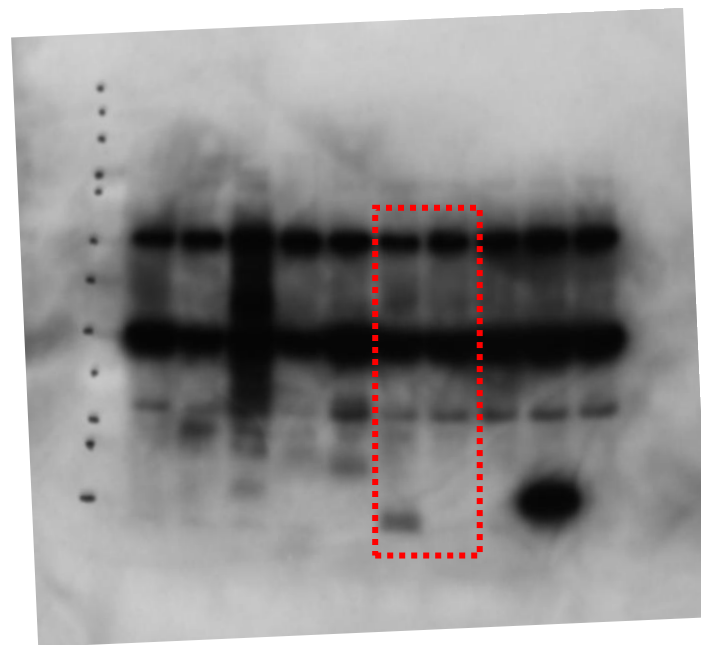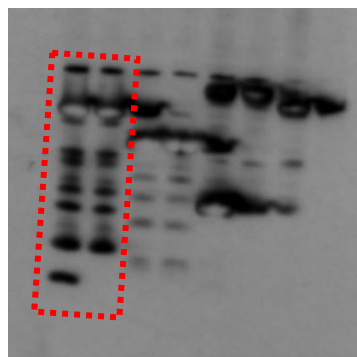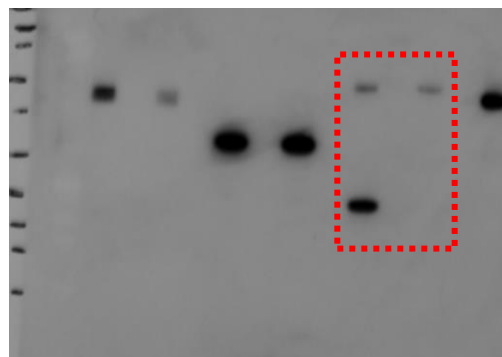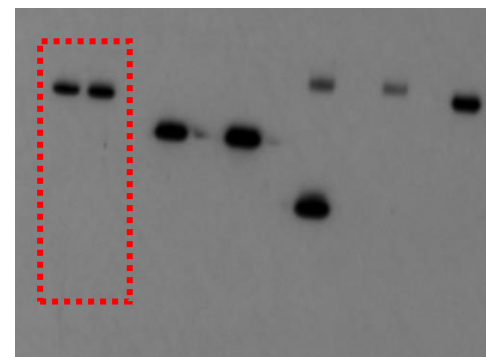

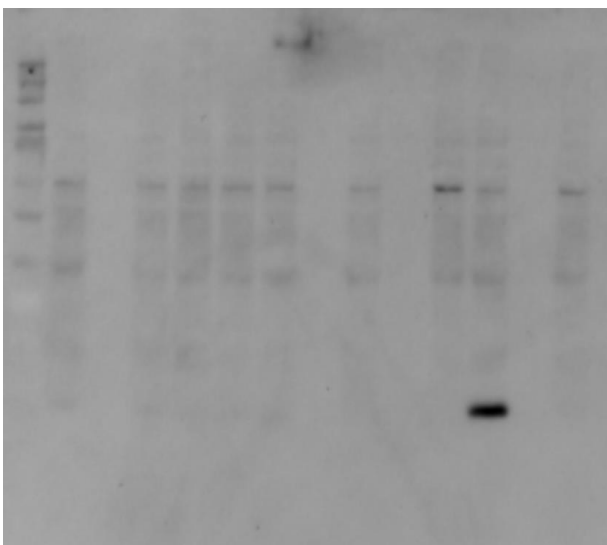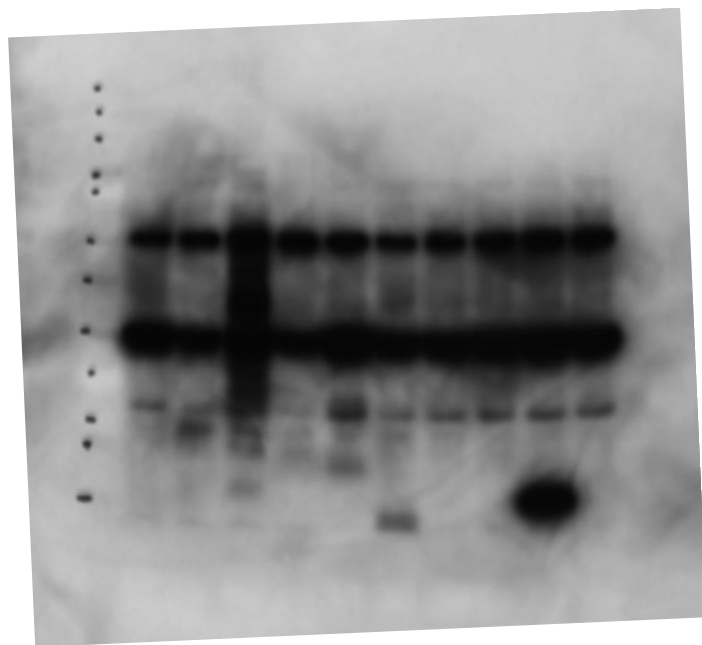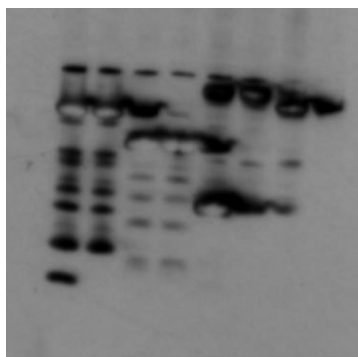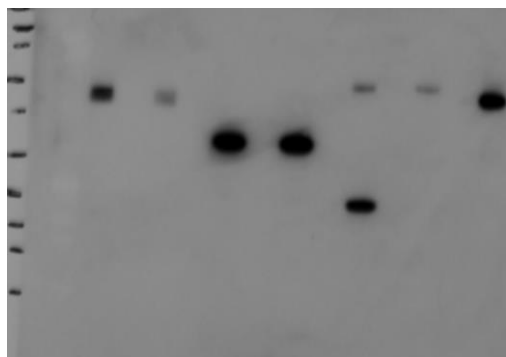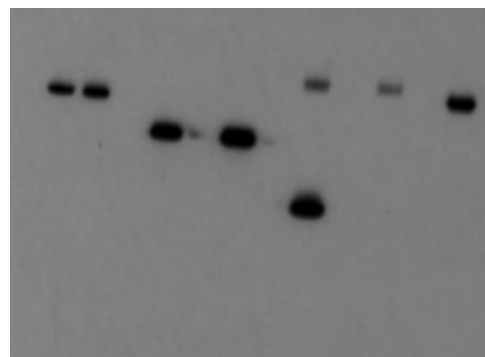

Supplement: Figure 6—figure supplement 2—source data 1. — The zipped folder includes (i) individual files for each blot, and (ii) a summary file showing all blots, with boxes to show the regions used in Figure 6—figure supplement 2. [file elife-73980-fig6-figsupp2-data1.zip › Source data summary Figure 6 Figure Supplement 2.pdf]

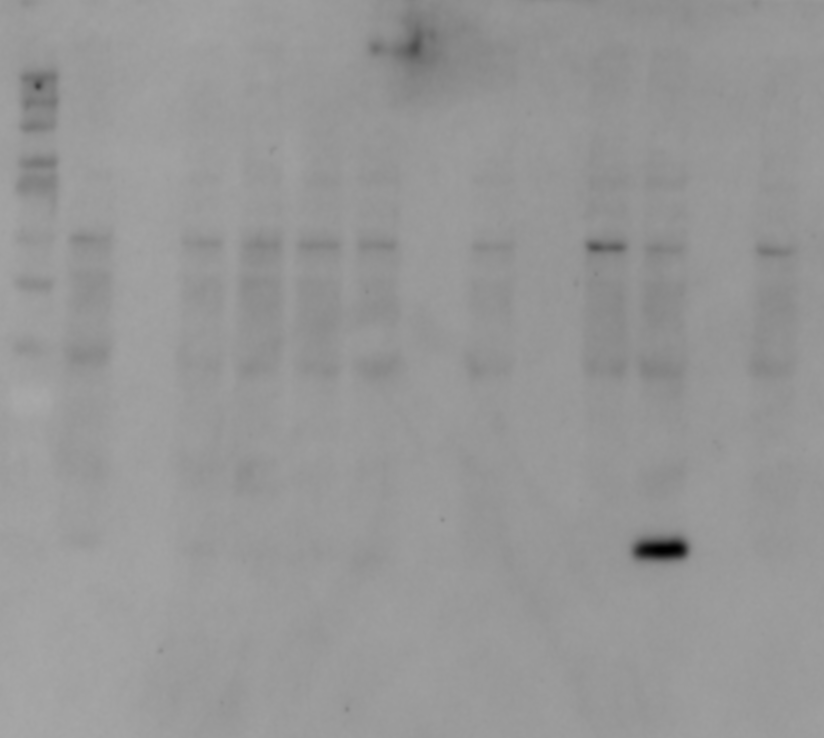

Supplement: Figure 6—figure supplement 2—source data 1. — The zipped folder includes (i) individual files for each blot, and (ii) a summary file showing all blots, with boxes to show the regions used in Figure 6—figure supplement 2. [file elife-73980-fig6-figsupp2-data1.zip › Source_data_Figure_6_S2A_left_panel.tif]

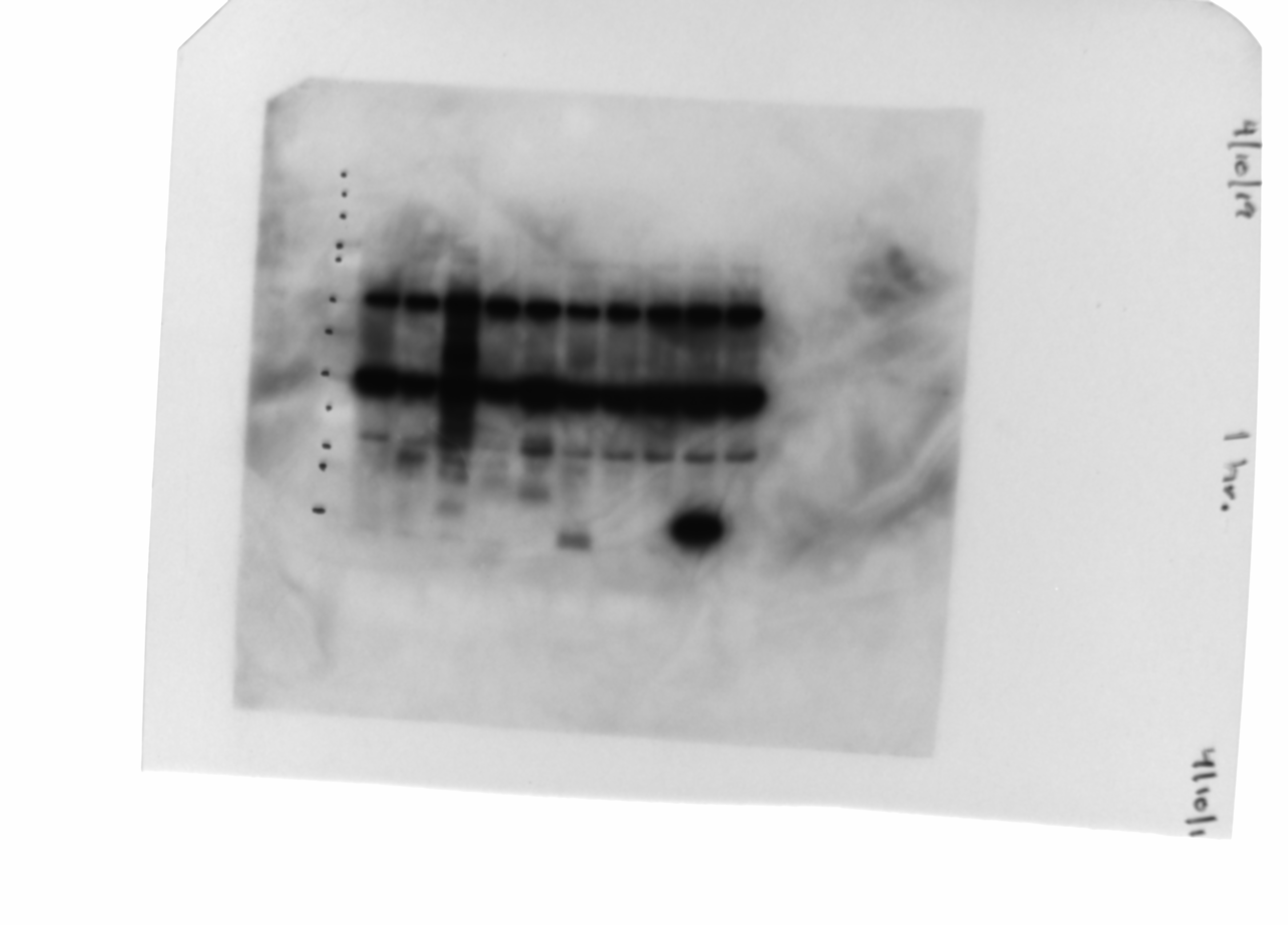

Supplement: Figure 6—figure supplement 2—source data 1. — The zipped folder includes (i) individual files for each blot, and (ii) a summary file showing all blots, with boxes to show the regions used in Figure 6—figure supplement 2. [file elife-73980-fig6-figsupp2-data1.zip › Source_data_Figure_6_S2A_right_panel.tif]

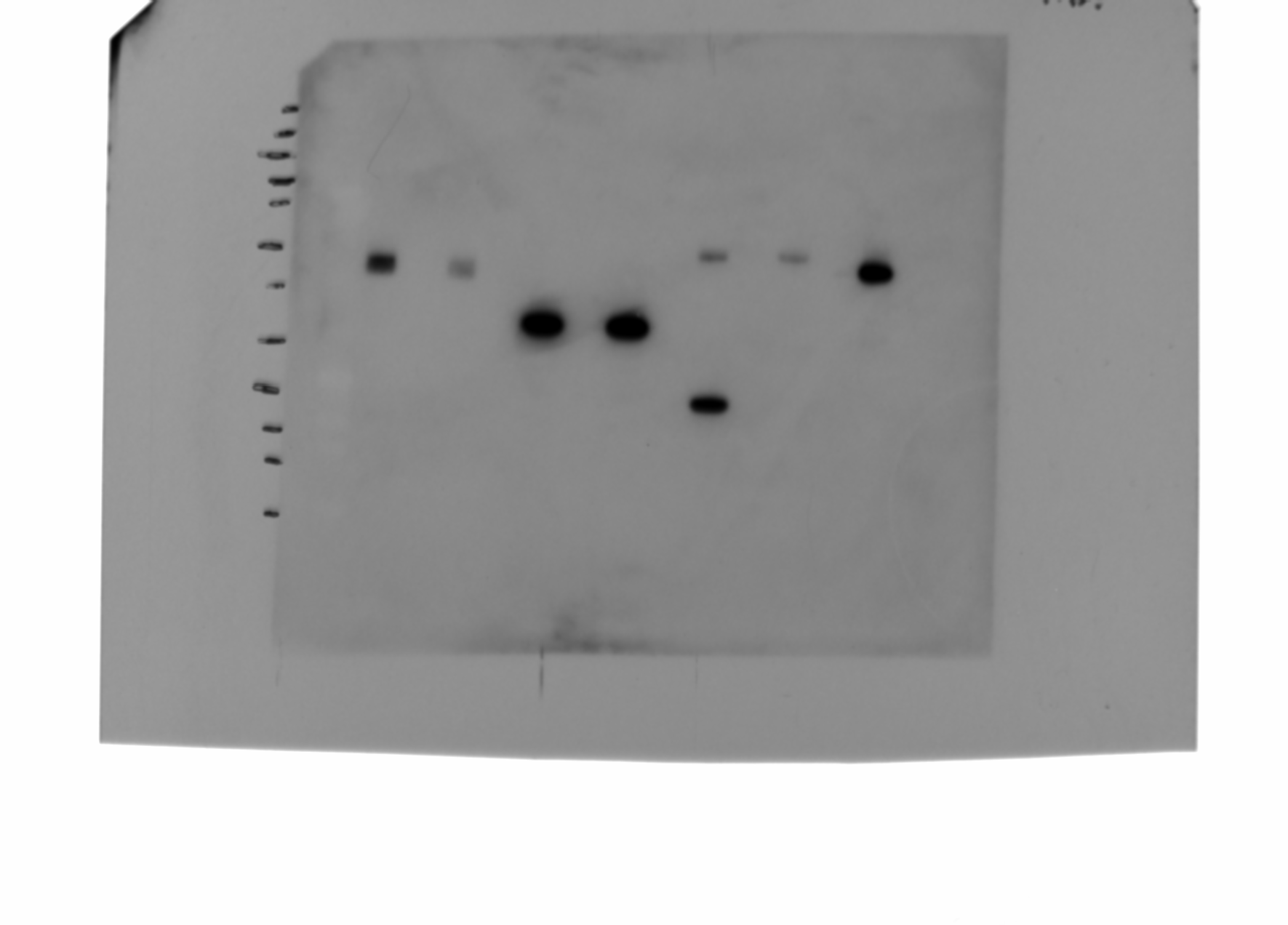

Supplement: Figure 6—figure supplement 2—source data 1. — The zipped folder includes (i) individual files for each blot, and (ii) a summary file showing all blots, with boxes to show the regions used in Figure 6—figure supplement 2. [file elife-73980-fig6-figsupp2-data1.zip › Source_data_Figure_6_S2B_left_panel.tif]

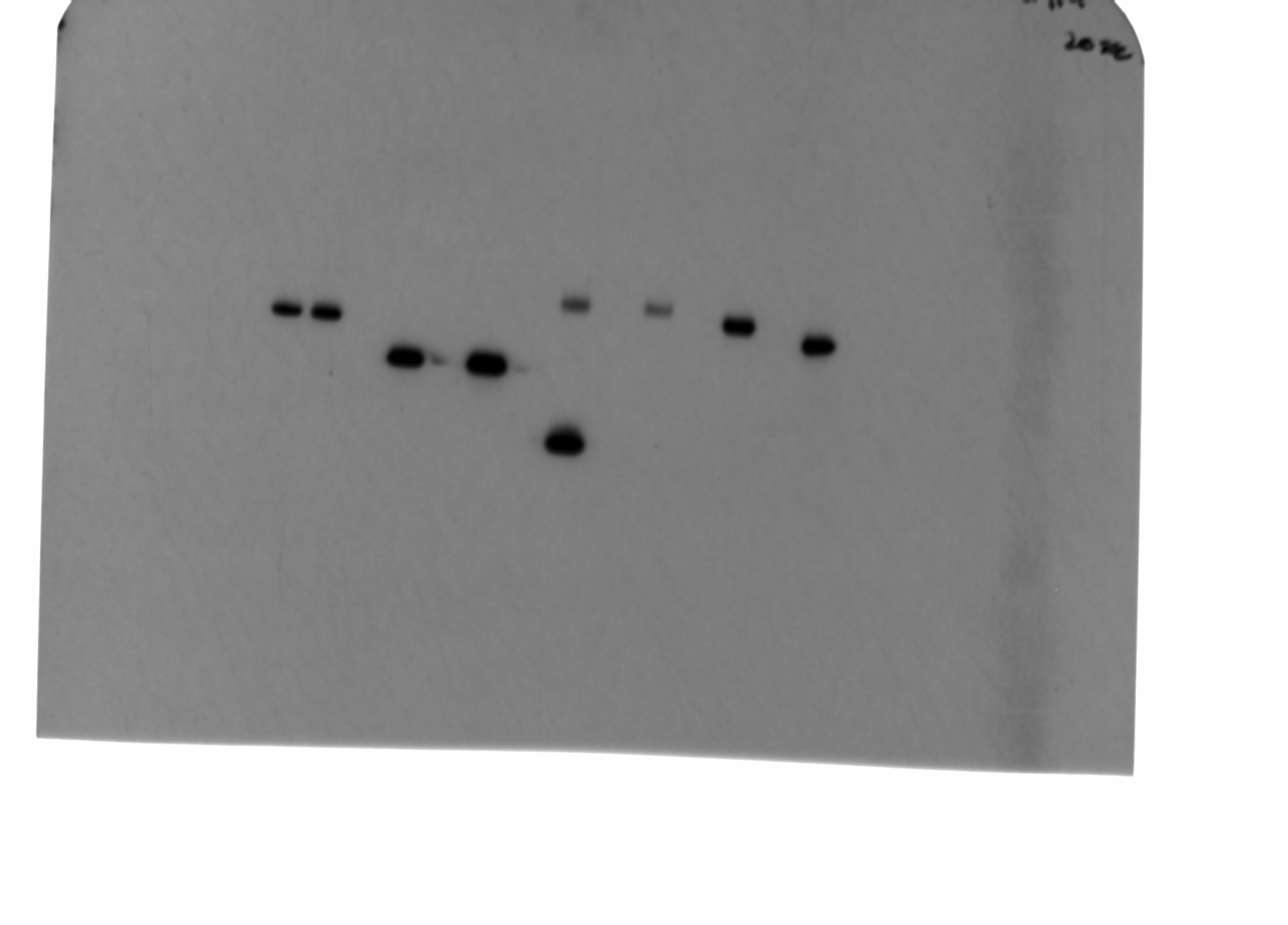

Supplement: Figure 6—figure supplement 2—source data 1. — The zipped folder includes (i) individual files for each blot, and (ii) a summary file showing all blots, with boxes to show the regions used in Figure 6—figure supplement 2. [file elife-73980-fig6-figsupp2-data1.zip › Source_data_Figure_6_S2B_middle_panel.tif]

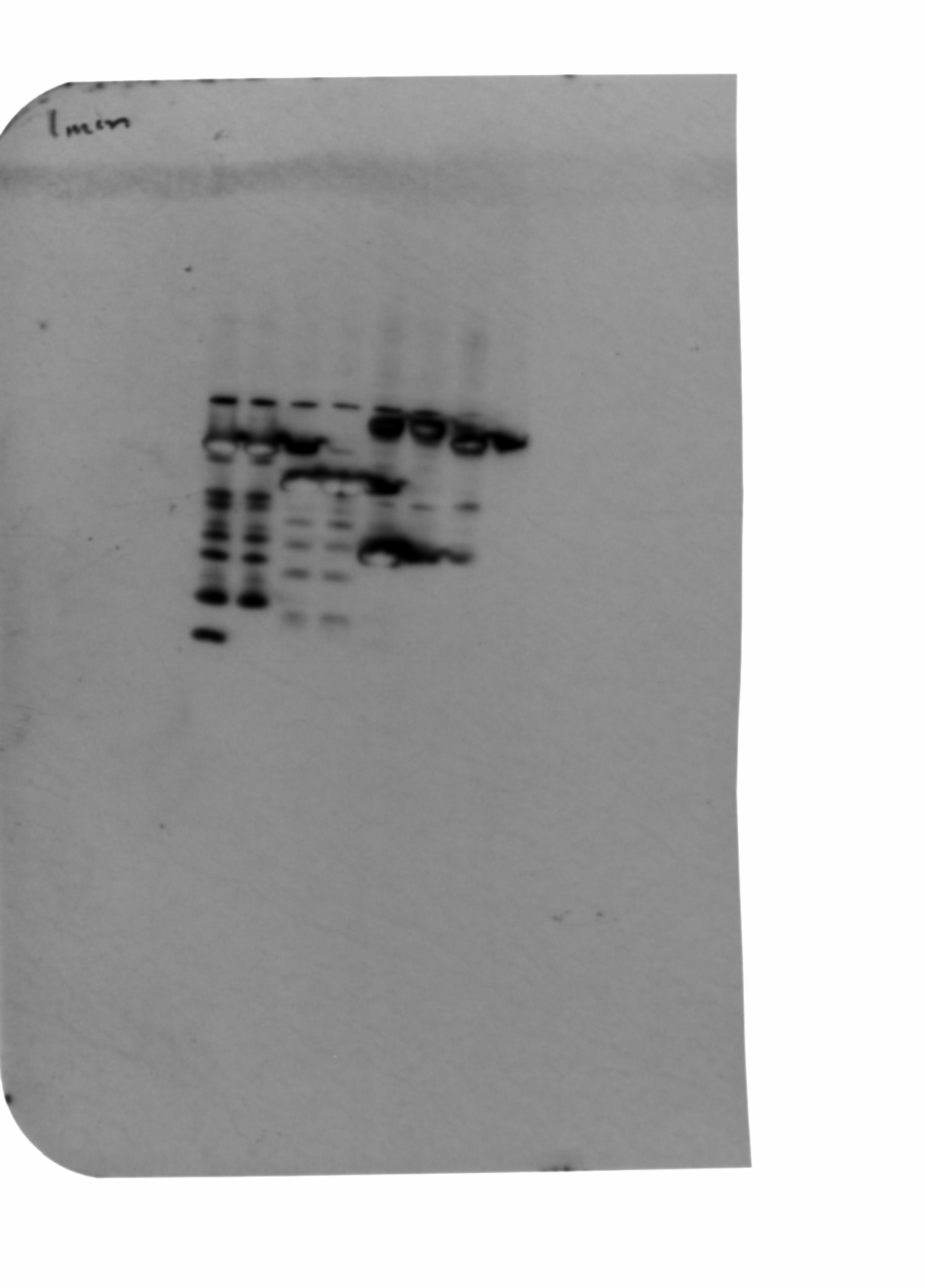

Supplement: Figure 6—figure supplement 2—source data 1. — The zipped folder includes (i) individual files for each blot, and (ii) a summary file showing all blots, with boxes to show the regions used in Figure 6—figure supplement 2. [file elife-73980-fig6-figsupp2-data1.zip › Source_data_Figure_6_S2B_right_panel.tif]
